# Coagulation Effect of Sugammadex as Determined by Thromboelastography in a Randomized Controlled Study of Surgical Patients

**DOI:** 10.7150/ijms.42563

**Published:** 2021-01-21

**Authors:** Hae Wone Chang, IL Ok Lee, Hyoseok Kang, Young Ju Won, Young-seob Lim

**Affiliations:** 1 Department of Anesthesiology and Pain Medicine, Eulji University Hospital, Seoul, Korea.; 2 Department of Anesthesiology and Pain Medicine, Korea University Guro Hospital, Seoul, Korea.

**Keywords:** Sugammadex, Coagulation, Thromboelastography, Laparoscopic surgery

## Abstract

**Introduction**: Sugammadex has been shown to be associated with prolongation of prothrombin time and activated partial thromboplastin time. However, it is not known whether it could be associated with enhancing postoperative hypocoagulation. The objective of this study was to analyze the effect of 4 mg/kg of sugammadex on thromboelastography (TEG) parameters in surgical patients.

**Methods:** After Institutional Review Board approval, a prospective double-blinded randomized controlled study was conducted between September 2016 and April 2017. Sixty adult patients scheduled for laparoscopic abdominal surgery were randomly allocated to receive either sugammadex 4 mg/kg (sugammadex group) or pyridostigmine 0.15 mg/kg in combination with glycopyrrolate 0.4 mg (control group) to reverse rocuronium-induced neuromuscular blockade at the completion of surgery. Blood samples were collected three time points; After the final suture of surgery (baseline) (T1), and at 10 min (T2) and 1 h (T3) after administration of the study drug. Whole blood was analyzed by TEG using TEG 5000 (Hemonetics Corp, Braintree, MA, USA). The primary endpoints were comparison of coagulation time (K, time to 20 mm clot amplitude), R (reaction time), alpha angle, and maximal amplitude (MA) between two groups.

**Results:** Coagulation time was significantly prolonged in sugammadex group after 10 min of the study drug administration compared to control group (mean value 1.3 ± 0.4 vs. 1.5 ± 0.4, P = 0.03). However, R, alpha angle and MA value were not different between two groups.

**Conclusions:** Sugammadex 4 mg/kg showed an increase in coagulation time in surgical patients. Physician should aware the potential enhancement of hypocoagulation by sugammadex in the setting of high risk of postoperative bleeding.

## Introduction

Sugammadex (Bridion^®^, Merck Sharp & Dohme B.V., Haarlem, the Netherlands), a chemically modified gamma-cyclodextrin is a highly selective binding agent for aminosteroidal neuromuscular blockers, including rocuronium and vecuronium 1.The structure of outer surface of sugammadex is composed of negatively charged polar hydroxyl groups, which strongly bind with the positively charged nitrogen molecule of rocuronium 2. Encapsulation of rocuronium by sugamadex reduces free plasma rocuronium, and concentration gradient promotes clearance of rocuronium molecule from neuromuscular junction. In this manner, sugammadex can provide more rapid and reliable recovery from neuromuscular block compared to traditionally used acetylcholinesterase inhibitors (pyridostigmine). Pyridostigmine has systemic cholinergic side effect, it must be used with anticholineric drug, glycopyrrolate. On the other hands, sugammadex rarely has biologic effects, and there is no need for anticholinergic drug; however, concerns have been raised regarding coagulation function as some cyclodextrin sulfates was known to bind to multiple coagulation factors. De Kam et al. reported that sugammadex caused a moderate (11-22%) increase in the prothrombin time (PT) and activated partial thromboplastin time (aPTT) in healthy volunteers 3. However, subsequent studies with cancer surgery, and orthopedic surgery failed to show increased coagulation times after use of sugammadex 4-6. Dirkmann et al. suggested that potential interaction between sugammadex and phospholipid would play key role in prolongation of coagulation time, which is possibly considered as *in vitro* action 7. Thromboelastography (TEG) traditionally has been used for hemostatic guidance for high risk of bleeding procedure. It measure shear elasticity of a coagulating whole blood using electro-mechanical transducer of movement of a torsion wire connected to the suspended pin. This mechanism enable TEG uniquely detect hypocoagulation state caused by massive blood loss as well as anticoagulant drug 8. Under the assumption that sugammadex has substantial anticoagulation activity, we hypothesized that TEG would reveal changes in parameters related to the enzymatic coagulation cascade 9. Therefore, we aimed to study if a dose of 4 mg/kg of sugammadex would affect perioperative coagulation time in patients undergoing abdominal laparoscopic surgery. We compared the change in K (coagulation time, time to 20 mm clot amplitude), R (reaction time), angle, and maximal amplitude (MA) in patients receiving sugammadex 4 mg/kg, with a combination of pyridostigmine and glycopyrrolate following laparoscopic surgery (control).

## Methods

Approval for this study was obtained from the Korea University Guro Hospital Institutional Review Board (KUGH16159). This clinical trial was registered with the UMIN Clinical Trials Registry (UMIN-CTR) (Unique ID: UMIN000024163, dated 26 September 2016; Principal Investigator: Dr. Chang). This study is in accordance with the reporting guidelines, defined by the EQUATOR Network. All patients were recruited from the Korea University Guro Hospital. Written informed consent was taken from patients who agreed to participate in the study on the day prior to surgery.

### Study population

Patients aged 19-65 years scheduled for elective abdominal laparoscopic surgery were included. All patients were classified as American Society of Anesthesiologists (ASA) class I-II. Patients were excluded from the study if they had neuromuscular diseases, hereditary coagulation defects, renal or hepatic dysfunction, known allergy to sugammadex, a history of hemorrhage or thrombosis within 30 days, expected difficult airway, abnormal coagulation parameters (PT internalization normalized ratio [PT-INR] > 1.5, or aPTT > 50 s), if they were on aspirin or anticoagulant medication, if they were pregnant or breastfeeding, or if they refused to participate.

### Study design, randomization and masking

This study was designed as a double-blind, randomized controlled trial. The investigators and patients were blinded to group allocation.

Independent coordinator performed allocation process through computer generated random number after enrollment. One investigator was aware of the allocated intervention. Group assignment was concealed in a sealed envelope and opened prior induction of anesthesia. Anesthesiologists knew the allocated group, but, they only followed the anesthetic protocol of our study, and did not engaged with the sampling of blood and performing TEG process. Based on group allocation, patients received either a combination of pyridostigmine 0.15 mg/kg and glycopyrrolate 0.4 mg or sugammadex 4 mg/kg to reverse the effect of rocuronium.

### Anesthesia protocol and Intervention

Anesthesia was induced with propofol, rocuronium, and 3% sevoflurane in oxygen using high fresh gas flows of 6-8 l/min. Remifentanil infusion was started at 0.05-0.2 μg/kg/min. Neuromuscular monitoring was performed at the adductor pollicis muscle using acceleromyography (TOF -Watch^®^ SX, Organon [Ireland] Ltd., Swords, Ireland) continuously with the time interval of 5 minutes until the end of anesthesia. A maintenance dose of rocuronium 0.1-0.2 mg/kg was administered for intraoperative neuromuscular blockade for maintain TOF 0-2 during the procedure. All patients received a crystalloid solution; colloids and blood products were not administered. The maintenance fluid was administered at 4-6 ml/kg/hr. The same anesthetic agents and techniques were used in all patients. After final suture of surgery, an investigator inserted 18-gauge intravenous cannula at forearm vein. The catheters were continuously flushed with 0.9% sodium chloride and contained no heparin. Baseline sample was obtained after discarding 8 ml of blood, corresponding to approximately twice the tubing volume, to avoid any contamination with sodium chloride. Sevoflurane inhalation was discontinued, and washout was performed using 100% oxygen at 10 l/min. Remifentanil infusion was stopped. When end-tidal concentration of sevoflurane was approach to 0.2 minimum alveolar concentration (MAC), and TOF monitoring was performed. After confirmation of appearance of the forth of twitch, a patients received either sugammadex 4 mg/kg (sugammadex group) or a combination of pyridostigmine 0.15 mg/kg and glycopyrrolate 0.4 mg (control group) for reversal of neuromuscular blockade according to group assignment.

After the patient recovered spontaneous breathing and consciousness, extubation was performed. Second sample was obtained after 10 minutes after the administration of the study drug. The patient was transferred to the post anesthetic care unit (PACU). Third sample was obtained at 1 h after administration of the study drugs in the PACU.

### Thromboelastography assays

A computerized thromboelastographic assay was performed using the TEG^®^ 5000 hemostasis analyzer (Haemonetics^®^, Braintree, MA, USA) with a disposable plastic cup and pin for native whole blood. An aliquot of 360 μl blood was placed in a TEG cup within 4 min after sampling. The TEG® analyzer was pre-warmed to 37°C. The variables recorded included (1) the reaction time (r, min), representing the rate of initial fibrin formation; (2) the coagulation time (K, min), representing the time until a fixed level of clot firmness or time in minutes from R until an amplitude of 20 mm; (3) the angle (α, degrees), which is closely related to K and represents the rate of clot formation determined by the upslope of the TEG tracing from the R-value; (4) the maximum amplitude (MA, mm), representing the maximum clot strength and depicted as the greatest width of the TEG tracing; and (5) the shear elastic modulus strength (G, dynes/cm^2^), a parametric measure of clot firmness expressed in metric units calculated from MA as follows:

G = (5000 × MA) / (100 - MA)

In addition to the conventional TEG parameters, we measured parameters of clot kinetics using a graph generated by TEG. The parameters of clot kinetics provided additional information on the kinetics of the coagulation cascade as they represent the more parametric measurements of clot propagation. The following variables were recorded: the maximum rate of thrombus generation (MRTG, dynes/cm^2^/s) was measured as the first derivate of the velocity of the increase in clot strength, commencing as G started to rise and ending after stabilization of clot strength. The information from this variable was equivalent to the information from the angle α. Time to maximum clot strength (TMRTG, min) is the time necessary to reach MRTG, which reflects the enzymatic contribution to clot formation. In addition, we determined the total thrombin time (TTG), which is the total positive area under the velocity curve, representing the total change in elastic resistance until clot strength stabilization occurs. MRTG and TTG were expressed using metric units of elastic resistance that accurately describe changes in clot strength. The TEG parameters were calculated by a person blinded to group assignment at the time of sampling. The primary outcomes were the change of coagulation time, R, angle alpha and MA between control and sugammadex group; Secondary outcomes were the change of G, MRTG, TMRTG, and TTG, and postoperative bleeding between two groups.

### Statistics

The primary endpoint of this study was change of coagulation time measured after administration of the pyridostigmine-glycopyrrolate combination or sugammadex.

We calculated a sample size of 26 patients for each group, based on data from a pilot study of six cases in each group as there were no previous studies available. In the pilot study, the mean and standard deviation value of coagulation time in the two pre-defined groups were 1.4 ± 0.5 and 1.8 ± 0.6 respectively. Thus, the effect size f of the two groups was assumed to be 0.72. A sample size of 26 patients was derived for each group, calculated using the Wilcoxon-Mann-Whitney test, a level of significance of 0.05, and a power of 0.8 (G power 3.1, Brunsbüttel, Germany). Allowing for possible dropouts, we enrolled 30 patients in each group (total of 60 patients).

Data were assessed for normal distribution of variance using normality plots and the Kolmogorov-Smirnov test. Student's t test was used to analyze the normally distributed continuous variables, and data was presented as mean and standard deviation (SD). The Fisher exact or χ^2^ test was used for categorical variables. All primary analyses of TEG data were performed on an intention to treat basis. Changes in TEG parameters over time were compared with baseline value in each group by mixed effect analysis of two ways factor with repeated measures, and multiple comparisons were performed. A p value < 0.05 was considered statistically significant. The analysis was performed using GraphPad Prism version 8.0.3 for Windows (GraphPad Inc., La Jolla, CA, USA).

## Results

**Figure 1:**
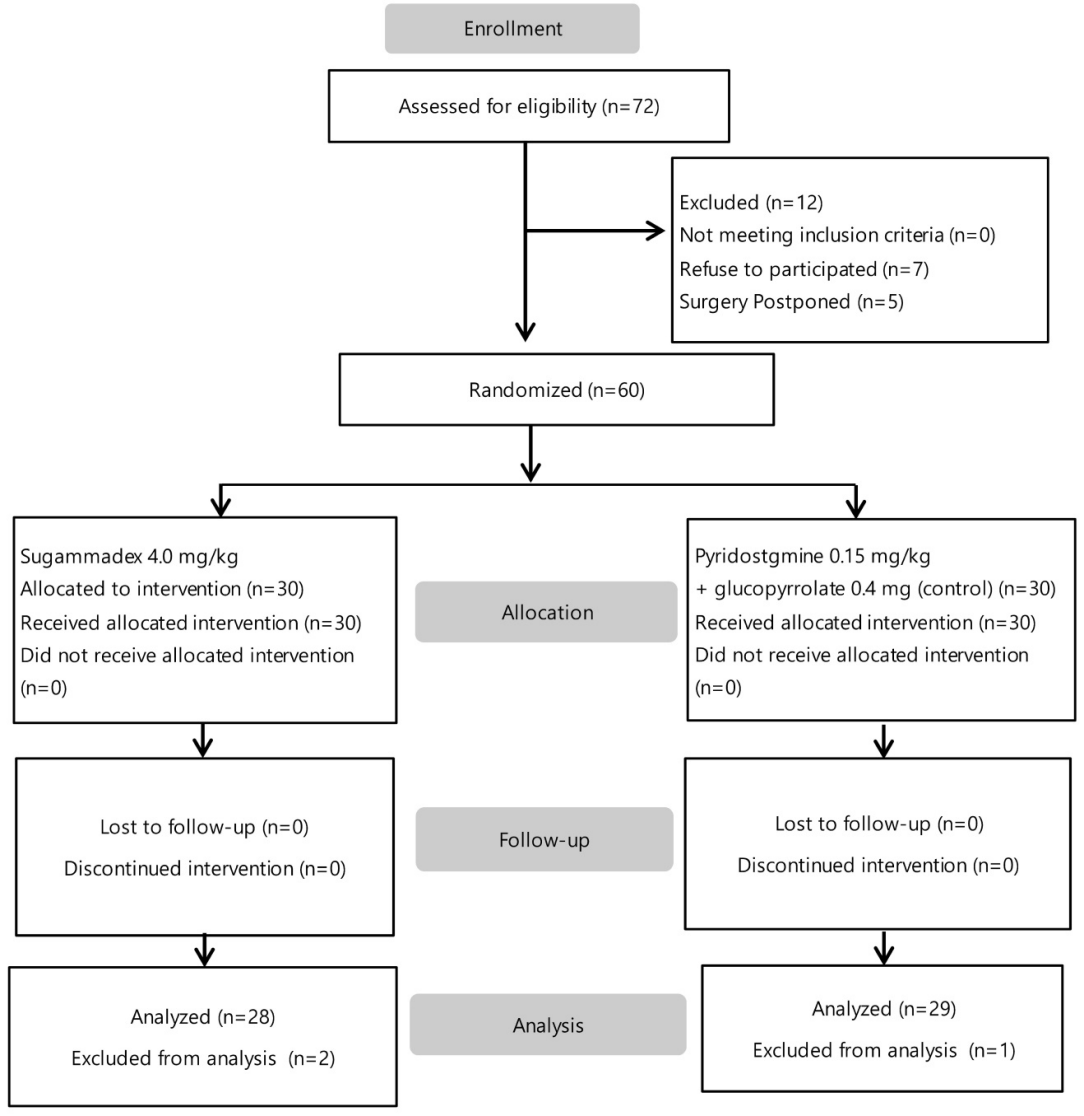
Flow chart of study.

Of 72 patients who were screened, 12 were excluded (7 refused to participate, and 5 surgery postponed). The enrolled 60 patients were assigned to one of two groups. Data from three patients (one in the control group and two in the sugammadex group) was excluded due to incomplete TEG graph during analysis. Fifty-seven patients were included in the final analysis (Figure 1). Baseline characteristics including age, sex, body weight, body mass index, duration of surgery and anesthesia, total fluid intake, and estimated blood loss were not different between groups (Table 1). The preoperative hemoglobin level, hematocrit (Hct), platelet and leukocyte counts, PT, aPTT, PT INR, and Creatinine (Cr) levels were not different between groups. All procedures were performed using a laparoscopic surgical technique. A crystalloid solution was administered to all patients; colloid solutions or blood products were not administered. The anesthetic technique and agents used were uniform.

Among the TEG parameters, mean value of K (coagulation time) was significantly prolonged in sugammadex group after 10 min after the study drug administration even though it was clinically within normal range (p = 0.03). P value of group factor was 0.02, and p value of interaction between time and group was 0.78. R time, G, TMRTG, and TTG were higher, and MRTG was lower in sugammadex group without statistical significance (Table 2). Angle and MA were not different between two groups. Within-group analysis revealed that the R time (p = 0.009) and TMRTG (p = 0.03) were significantly lower in the control group compared to baseline values at the time of 1 h after the administration of study drug whereas the differences were not shown in sugammadex group. Postoperative 24 hour bleeding volume between control and sugammadex group was not different. There were no significant differences of postoperative 24 hour Hb levels, PT and aPTT between groups (Table 1).

## Discussion

Our present randomized controlled study demonstrated several points. Firstly, we found increase of coagulation time following administration of sugammadex, approximately 6.8% compared to the value of baseline, and 17.2% compared to control group. Coagulation time, speed of clot propagation might be affected by sugammadex mediated coagulation change. However, R, angle and MA were not changed. Nielsen et al. studied specific coagulation factor deficiency associated change of TEG parameters 10. In their study, R value was significantly decreased with each value of increased in FX activity throughout the activity range, while angle value significantly increased with each increase in FX activity, with a plateau observed between 50% and 100% activity. Therefore, effect of 4mg/kg of sugammdex mediated Factor Xa activity would not change of these parameters. Furthermore, TEG can only show overall hemostatic state, hypercoagulability in immediate postoperative period may offset the effect of increase of coagulation inhibitor. Topal et al. reported that high intra-abdominal pressures caused by pneumoperitoneum during laparoscopy increases postoperative MA, and angle, and decrease R time at 30 minutes after operation 11. Secondly, we did not use citrated sample, as a result, the cause of prolongation of coagulation time was not considered with the same logic of PT and aPTT in previous studies. The observed effects of sugammadex in this study were unlikely due to the binding of phospholipids contained in coagulation assays. Third, we tested sample at the time of 10 minutes after the administration of 4 mg/kg of sugammadex in the setting of moderate level of neuromuscular block. Carron et al. reported a sugammadex dose of 2 and 4 mg/kg prolonged rotational thromboelastometry (ROTEM™) clotting time by 7.7 and 10.7% compared with baseline value in morbidly obese patients undergoing laparoscopic sleeve gastrectomy 12. In their study, greatest increase in coagulation time was found at 3 minutes after administration of sugammadex, and it was positive relationship with total dose of sugammadex. In this study, we observed the change in K time after 10 min following administration of sugammadex, we may have missed the peak effect of sugammadex.

De Kam et al. reported a 5-17% increase in PT and aPTT following sugammdex dose of 4 mg/kg. 13. An *in vitro* study demonstrated more prolonged PT and aPTT values compared to *in vivo* studies. When sugammadex was added to normal human plasma at a concentration of 200 µg/ml, PT and aPTT increased by 20 - 30%. The mechanism of prolonged coagulation time by sugammadex was proposed from inhibition of factor Xa activity through either common pathway or combined effect with factor Xa formation by intrinsic route 3. Under the assumption that sugammadex has substantial anticoagulation activity, we hypothesized that a viscoelastic test would reveal changes in parameters related to the enzymatic coagulation cascade. Previous reported *in vitro* TEG study with orthopaedic patients, higher sugammadex concentration resulted in a linear increase in R and K time along with decreased the angle and MA, suggest dose dependent enhanced hypocoagulation 14. In an observational clinical study in cancer patients, there was no evidence of additional postoperative bleeding, with no increase in clotting time after the use of sugammadex for reversal of muscle relaxants 4. Rahe-Meyer et al. reported that in 1184 patients undergoing hip surgery and joint replacement surgery with thromboprophylaxis, sugammadex 4 mg/kg was associated with an increase in PT and aPTT by 3-5.5% 5; however, they did not find an increased risk of bleeding in the sugammadex and anticoagulant therapy group compared to usual care and anticoagulation therapy. As sugammadex has a short elimination half-life (approximately 1.8 h), the prolongation was limited to less than 1 h in these studies. A clinical study including 40 patients undergoing septoplasty reported that sugammadex increased postoperative bleeding, but the amount of bleeding was small, and indeed the PT and aPTT values were similar at 2 h after surgery 15. While the mechanism of anticoagulation is unknown, Dirkmann et al. demonstrated that increased clotting time was observed only after administration of free sugammadex, and not after administration of the sugammadex-rocuronium complex using *in vitro* rotational thromboelastometry 7. They speculated that sugammadex may have an affinity for binding with phospholipid in the absence of rocuronium. Van der Waals forces during complexation procedures of gamma cyclodextrine and phospholipid might affect *in vitro* coagulation studies that use phospholipids 16. The penetration of the hydrophobic part of the molecule into the cavity leads to dehydration of the guest molecule and indicates that van der Waals forces and hydrophobic interactions are involved in the binding of sugammadex.

The clotting cascade requires the assembly of protease-cofactor complexes on membranes with exposed anionic phospholipids 17. An anionic lipid contains a negatively charged carboxyl group, which enables calcium to form bridges with γ carboxyglutamic acid (gla) domain of key coagulation factors. A previous study has revealed that carboxyethylated γ cyclodextrin greatly enhances the transfer of a variety of hydrophobic phospholipid derivatives from vesicles to cultured cells 18. Therefore, sugammadex may generate possible Van der Waals forces or cause a hydrophobic interaction with anionic phospholipid, which may affect the interaction between clotting proteins and membrane surfaces *in vitro* study 19.

The pharmacodynamic interaction between sugammadex and other anticoagulant drugs were investigated in several studies. A clinical study demonstrated no increase in anti-Xa activity after sugammadex 4 mg/kg or 16 mg/kg following pretreatment with enoxaparin or unfractionated heparin in healthy subjects 20. In healthy volunteers who received sugammadex 4 mg/kg and oral aspirin 75 mg, aPTT was increased by 3.5 s after sugammadex administration, and by 7 s after administration of the aspirin and sugammadex combination. There was a 20% increase in bleeding time after the combined administration of sugammadex and aspirin compared with aspirin alone 3. Davidson et al. reported that nonsteroidal anti-inflammatory drugs (NSAIDs) with anticoagulant therapy increased the risk of bleeding to the same extent as aspirin combined with an anticoagulant, and it was not associated with the duration of NSAID therapy 21. Therefore, further study would be needed for elucidate the possible mechanism of anticoagulation action by sugammadex in various coagulation studies as patients who anticipate to undergoing surgery on antiplatelet or anticoagulant drug may have further risk of bleeding.

Our study has several limitations. Firstly, we did not measure baseline TEG before surgery, and various anesthetic and surgical factors may account for the results. However, we attempted to minimize the influence of these factors. We recruited patients who underwent laparoscopic surgery associated with minimal or moderate hemorrhage. We also maintained the same anesthetic technique and used only a crystalloid solution in both groups. Besides, the baseline values of data were obtained at the same depth of anesthesia after the completion of surgery, which were comparable between both groups. Secondly, we only tested the effects of 4 mg/kg dose of sugammadex. Different doses of sugammadex of 2mg/kg or 16 mg/kg would be necessary for confirming prolongation effect of coagulation time by sugammadex. Thirdly, this is single center study, and sample size is small, so large number of patients and different surgical setting may be required to elucidate the potential effect of sugammadex on coagulation. Finally, our results indicated anticoagulant effect in the sugammadex group compared to pyridostigmine group. However, we did not measure specific blood coagulation factors. Therefore, we could not determine exact mechanism of anticoagulation action of sugammadex.

In summary, administration of sugammadex 4 mg/kg to surgical patients with no history of coagulation abnormalities showed prolongation of coagulation time in TEG. Recently, plasma enhanced chemical vapor deposition (PECVD) coatings on TEG cup and pin has been proposed to compare the precise effects of activators and inhibitors on the intrinsic coagulation cascade in platelet-poor plasma 22. Therefore, a new technology applied to TEG can help understanding of coagulation effects of drug in the future.

## Figures and Tables

**Table 1:** Patient Characteristics. Values are expressed as mean (SD)

	Control (n = 30)	Sugammadex (n = 30)
Age (y)	45 (8.6)	45 (11.9)
Sex (Male/Female)	14/16	17/13
Body weight (kg)	67.4 (13.2)	63.8 (12.5)
Height (cm)	166.9 (6.7)	163.3(8.7)
BMI (kg/m^2^)	24.1 (4.3)	23.7 (3.1)
Hb (g/dl)	13.4 (1.8)	13.5 (1.8)
Hct (%)	40.2(4.5)	40.5 (4.8)
WBC (10^3^/μl)	6.5(2.4)	5.9(1.2)
Cr (mg/dl)	0.7(0.1)	0.7(0.2)
PT (s)	12.6(0.6)	12.7 (0.4)
PT INR	0.97(0.05)	0.97 (0.04)
aPTT (s)	33.3(3.2)	33.9 (3.1)
Platelet (10^3^/μl)	254 (98.1)	247.2 (58.3)
Duration of surgery (min)	89 (57)	99 (72)
Duration of anesthesia (min)	134(60)	140 (81)
Fluid intake (ml)	674(625)	660 (685)
Estimated blood loss (ml)	309 (54)	408 (76)
Postoperative 24-hour Hb (g/dl)	12.2 (1.6)	12.0 (2.1)
Postoperative 24-hour PT INR	1.1 (0.05)	1.2 (0.06)
Postoperative 24-hour APTT	34.5 (4.3)	35.2 (5.2)
Postoperative 24-hour bleeding volume	36 (70)	40 (10)

BMI=body mass index; Hb=hemoglobin; Hct=hematocrit; WBC=white blood cell; Cr= creatinine; PT=prothrombin time; PT INR=PT internalization normalized ratio; aPTT= activated partial thromboplastin time.

**Table 2:** Change of thromboelastography parameters of patients receiving sugammadex or a combination of pyridostigmine and glycopyrrolate (control). Values are expressed as mean (SD) and 95% confidence interval.

Analysis	Control (n = 29)			Sugammadex (n = 28)		
	Mean (SD)			Mean (SD)		
	T1	T2	T3	T1	T2	T3
R (min) 4-8	4.7(1.5)	4.0(1.3)	3.8(1.2)**	4.7(1.9)	4.2(2.0)	4.2(2.6)
K (min) 1-4	1.5(0.4)	1.3(0.4)	1.5 (0.3)	1.5(0.4)	1.5(0.4)†	1.6(0.6)
Angle(degrees) 47-74	69.8(5.4)	70.9(5.7)	69.1(7.8)	69.9(6.0)	69.9(5.2)	68.8(7.7)
MA (min) 55-73	67.7(5.3)	69.4(4.1)	69.6(4.7)	67.2(5.8)	67.6(4.9)	67.3(4.5)
G (dynes/cm^2^)^-1^) 3.2-7.1	11.1(2.9)	11.8(2.6)	11.7(3.1)	10.6(3.0)	10.7(2.5)	10.6(2.3)
MRTG (mm/min) 9-21	15.3(4.3)	16.7(4.9)	15.9(3.7)	14.8(3.8)	14.6(3.4)	14.8(2.8)
TMRTG (min) 4-11	5.7(1.8)	4.9(1.6)	4.8(1.5)*	5.8(2.1)	5.4(2.5)	5.3(3.2)
TTG (mm/min) 632-861	825(68)	846(59)	843(57)	812(72)	818(63)	820(58)

T1=baseline before drug administration; T2=10 min after administration of the study drug; T3=1 hour after administration of the study drug; R=reaction time to clot formation; K=time to achieve a clot strength of 20 mm amplitude; Angle= rate of clot growth; MA= maximum amplitude of clot strength; G=shear elastic modulus strength; MRTG=maximum rate of thrombus generation; TMRTG=time to maximum thrombus generation; TTG=total thrombin time; *p < 0.05 compared with baseline value, **p < 0.01 compared with baseline value. †p < 0.05 compared with control.
